# *d*-Neighborhood system and generalized F-contraction in dislocated metric space

**DOI:** 10.1186/s40064-015-1095-3

**Published:** 2015-07-22

**Authors:** P Sumati Kumari, Kastriot Zoto, Dinesh Panthi

**Affiliations:** Department of Mathematics, K.L. University, Vaddeswaram, Andhra Pradesh India; Department of Mathematics & Computer Science, Faculty of Natural Sciences, University of Gjirokastra, Gjirokastra, Albania; Department of Mathematics, Valmeeki Campus, Nepal Sanskrit University, Exhibition Road, Kathmandu, Nepal

**Keywords:** *d*-Metric space, *d*-Neighborhood system, $\mathcal {G}F$-Contraction., 47H10, 54H25

## Abstract

This paper, gives an answer for the Question 1.1 posed by Hitzler (Generalized metrics and topology in logic programming semantics, 2001) by means of "Topological aspects of d-metric space with d-neighborhood system". We have investigated the topological aspects of a d-neighborhood system obtained from dislocated metric space (simply *d*-metric space) which has got useful applications in the semantic analysis of logic programming. Further more we have generalized the notion of F-contraction in the view of d-metric spaces and investigated the uniqueness of fixed point and coincidence point of such mappings.

## Background

Metrics appear everywhere in Mathematics: Geometry, Probability, statistics, coding theory, graph theory, pattern recognition, networks, computer graphics, molecular biology, theory of information and computer semantics are some of the fields in which metrics and/or their cousins play a significant role. The notion of metric spaces introduced by Frechet (1906), is one of the helpful topic in Analysis. Banach (1922) proved a fixed point theorem for contraction mapping in a complete metric space. The Banach contraction theorem is one of the primary result of functional analysis. After Banach contraction theorem, huge number of fixed point theorems have been established by various authors and they made different generalizations of this theorem.

Matthews (1985) generalized Banach contraction mapping theorem in dislocated metric space. Hitzler (2001) introduce the notion of dislocated metric (d-metric) space and presented variants of Banach contraction principle for various modified forms of a metric space including dislocated metric space and applied them to semantic analysis of logic programs. Hitzler (2001) has applied fixed point theorems for self maps on dislocated metric spaces, quasi dislocated metric spaces, generalized ultra metric spaces in his thesis “Generalized Metrics and Topology in Logic Programming Semantics”. In this context, Hitzler raised some related questions on the topological aspects of dislocated metrics.

Recently, Sarma and Kumari (2012) initiated the concept of d-balls and established topological properties on d-metric space. In the context of d-metric space, many papers have been published concerning fixed point, coincidence point and common fixed point theorems satisfying certain contractive conditions in dislocated metric space (see Karapinar and Salimi 2013; Kumari et al. 2012a, b; Zoto et al. 2014; Ahamad et al. 2013; Ren et al. 2013) which become an interesting topic in nowadays.

Of late several weaker forms of metric are extensively used in various fields such as programming languages, qualitative domain theory and so on.

Motivated by above, we give an answer for the Question 1.1 posed by Hitzler, further more we discuss some topological properties in d-neighborhood system obtained from dislocated metric space. Moreover, we generalize the notion of F-contraction initiated by Wardowski (2012) and we prove fixed point theorem. Our established results generalize similar results in the framework of dislocated metric space. Further more, we provide coincidence theorem in the setting of d-neighborhood systems.

## Preliminaries and notations

First, we collect some fundamental definitions, notions and basic results which are used throughout this section. For more details, the reader can refer to Hitzler (2001).

### **Definition 2.1**

Let *X* be a set. A relation $$<\!\!\!\!\!\circ \subseteq X\times \mathcal {P}(X)$$ is called a *d-membership relation* (on *X*) if it satisfies the following property for all $$x\in X$$ and $$A,B\subseteq X:$$$$x<\!\!\!\!\!\circ \, A$$ and $$A\subseteq B$$ implies $$x<\!\!\!\!\!\circ \, B$$.

### **Definition 2.2**

Let *X* be a set, let $<\!\!\!\!\!\circ$ be a *d*-membership relation on *X* and let $\mathcal {U}_{x}\ne \phi$ be a collection of subsets of *X* for each $x\in X$. We call $(\mathcal {U}_{x},<\!\!\!\!\!\circ \, )$ a *d*-$neighborhood \ system$ for *x* if it satisfies the following conditions.

(i) If $$U\in \mathcal {U}_{x},$$ then $$x<\!\!\!\!\!\circ \, U$$
(ii) If $U,V\in \mathcal {U}_{x},$ then $U\cap V\in \mathcal {U}_{x}$
(iii) If $U\in \mathcal {U}_{x},$ then there is a $V\subseteq U$ with $V\in \mathcal {U}_{x}$ such that for all $y<\!\!\!\!\!\circ \, V$ we have $U\in \mathcal {U}_{y}$
(iv) If $U\in \mathcal {U}_{x},$ and $U\subseteq V,$ then $V\in \mathcal {U}_{x}.$

Each $U\in \mathcal {U}_{x}$ is called a *d*-neighborhood of *x*. Finally, let *X* be a set and $<\!\!\!\!\!\circ$ be a *d*-membership relation on *X* and, for each $x\in X$, let $(\mathcal {U}_{x},<\!\!\!\!\!\circ \, )$ be a *d*-neighborhood system for *x*. Then $(X,\mathcal {U},<\!\!\!\!\!\circ \, ) \, (or \ simply \ X)$ is called a *d*-topological space, where $\mathcal {U}=\{\mathcal {U}_{x}/x\in X\}.$

### **Proposition 2.3**

*Let X be a nonempty set. A distance on X is a map*$d: X \times X \rightarrow [0, \infty ).$*A pair* (*X*, *d*) *is known asdislocated metric space* (Simply *d*-metric space) *ifdsatisfies the following conditions*

- ($$d_1$$) $$d(x, y) $$ = 0 $$\implies x= y$$
- ($$d_2$$) $$d(x, y)= d(y, x)$$
- ($d_3$) $d(x, z) \le d(x, y) + d (y, z)$*for allx*, *y*, *zinX*

If $x\in X$ and $\epsilon >0,$ the set $\mathcal {B}_{\epsilon }(x)=\{y/y\in X$ and $d(x,y)<\epsilon \}$ is called the ball with center at *x* and radius $\epsilon .$

### **Proposition 2.4**

*Let*$(X,\varrho )$*be ad-metric space. Define thed-membership relation*$<\!\!\!\!\!\circ$*as the relation*$\{(x,A)/$*there exists*$\epsilon >0$*for which*$\mathcal {B}_{\epsilon }(x)\subseteq A\}.$*For each*$x\in X,$*let*$\mathcal {U}_{x}$*be the collection of all subsetsA* of *Xsuch that*$x<\!\!\!\!\!\circ \, A$*. Then*$(\mathcal {U}_{x},<\!\!\!\!\!\circ \, )$* is ad-neighborhood system forx; for each*$x\in X.$

### **Definition 2.5**

Let $(X,\mathcal {U},<\!\!\!\!\!\circ \, )$ be a *d*-topological spaces and let $x\in X.$ A net $(x_{\gamma })$*d*-converges to $x\in X$ if for each *d*-neighborhood *U* of *x* we have that $x_{\gamma }$ is eventually in *U*, that is, there exists some $\gamma _{0}$ such that $x_{\gamma }\in U$ for each $\gamma >\gamma _{0}.$

### **Definition 2.6**

Let $(X,\varrho )$ be a *d*-metric space and let $(X,\mathcal {U},<\!\!\!\!\!\circ \, )$ be a *d*-topological spaces as in Proposition 2.4. Let $(x_{n})$ be a sequence in *X*. Then $(x_{n})$ converges in $(X,\varrho )$ if and only if $(x_{n})$*d*-converges in $(X,\mathcal {U},<\!\!\!\!\!\circ \, ).$

### **Definition 2.7**

Let *X* and *Y* be *d*-topological spaces and let $f:X\rightarrow Y$ be a function. Then *f* is *d*-continuous at $x_{0}\in X$ if for each *d*-neighborhood *V* of $f(x_{0})$ in *Y* there is a *d*-neighborhood *U* of $x_{0}$ in *X* such that $f(U)\subseteq V.$ We Say *f* is *d*-*continuous* on *X* if *f* is *d*-*continuous* at each $x_{0} \in X.$

### **Theorem 2.8**

*LetXandYbed-topological spaces and let*$$f:X\rightarrow Y$$*be a function. Thenfis ad-continuous if and only if for each net*$$(x_{\gamma })$$*inXwhichd-converges to some*$$x_{0} \in X,$$$$(f(x_{\gamma }))$$*is a net inYwhichd-converges to*$$f(x_{0})\in Y.$$

### **Proposition 2.9**

*Let*$(X,\varrho )$*and*$(Y,\varrho ^{\prime })$*bed-metric spaces, let*$f:X\rightarrow Y$*be a function and let*$(X,\mathcal {U},<\!\!\!\!\!\circ \, )$*and*$(Y,\mathcal {V},<\!\!\!\!\!\circ ^{\prime })$*be thed-topological spaces obtained from*$(X,\varrho )$*, respectively*$(Y,\varrho ^{\prime })$*as in Proposition 2.4. Thenf* is *d-continuous at*$x_{0} \in X$*if and only if for each*$\epsilon >0$*there exists a*$\delta >0$*such that*$f(\mathcal {B}_{\delta }(x_{0}))\subseteq \mathcal {B}_{\epsilon }(f(x_{0})).$

### **Definition 2.10**

Let $(X,\varrho )$ be a *d*-metric space, let $f:X\rightarrow X$ be a contraction with contractivity factor $\gamma$ and let $( X,\mathcal {U},<\!\!\!\!\!\circ \, )$ be the *d*- topological space obtained from *d*-metric $(X,\varrho )$ as in Proposition 2.4. Then *f* is *d*-continuous.

## Topological aspects of *d*-metric space with *d*-neighborhood system

The following question was put forth in Hitzler Thesis.

*(Question 1.1). Question: Is there a reasonable notion of d-open set corresponding to the notions of d-neighborhood, d-convergence and d-continuity.*

We provided an answer for the above open question by constructing below theorems.

### **Theorem 3.1**

*Let*$( X,\mathcal {U},<\!\!\!\!\!\circ \, )$*be ad-topological space. Define*$\mathfrak {J}=\{V/$*for each*$x\in V \, {{there \, exists}} \, A\in \mathcal {U}_{x} \,  {{such \, that}}\, A\subset V \}$*. Then*$\mathfrak {J}$*is a topology onX*.

### *Proof*

Clearly $\mathfrak {J}$ contains *X* and $\emptyset .$

Let $\{V_{\alpha }\}$ be an indexed family of non-empty elements of $\mathfrak {J}$.

Let $x\in \cup V_{\alpha }$ which implies that $x\in V_{\alpha }$ for some $\alpha.$

Thus there exists $A\in \mathcal {U}_{x}$ such that $A\subset V_{\alpha }\subset \cup V_{\alpha }$. Which implies that $\cup V_{\alpha }\in \mathfrak {J}.$

Let $\{ V_{\alpha _{i}} \}^{n}_{i=1}$ be any finite intersection of elements of $\mathfrak {J}.$

 We have to prove that $\cap ^{n}_{i=1}V_{\alpha _{i}}\in \mathfrak {J}.$ To obtain this, first we prove that if $G_{1},G_{2}\in \mathfrak {J}$ then $G_{1}\cap G_{2}\in \mathfrak {J}.$ Let $x\in G_{1}\cap G_{2}$.

Which implies that $x\in G_{1}$ and $x\in G_{2}$ then there exists $A_{1}\in \mathcal {U}_{x} \, {\text{such that}}\, A_{1}\subset G_{1}$ and there exists $A_{2}\in \mathcal {U}_{x} \, {\text{such that}}\,\, A_{2}\subset G_{2}.$

Which implies $A_{1}\cap A_{2}\in \mathcal {U}_{x}$ and $A_{1}\cap A_{2}\subset G_{1}\cap G_{2}$.

Thus $$ G_{1}\cap G_{2}\in \mathfrak {J}.$$ Hence by induction, we get $$\cap ^{n}_{i=1}V_{\alpha _{i}}\in \mathfrak {J}.$$$$\square $$

### **Definition 3.2**

Let $( X,\mathcal {U},<\!\!\!\!\!\circ \, )$ be a *d*-topological space and $A \subseteq X$ be a *d*-*open* if for every $x\in A$ there exists $\mathcal {U}\in \mathcal {U}_{x}\ni \mathcal {U}\subset A.$

### **Definition 3.3**

Let $( X,\mathcal {U},<\!\!\!\!\!\circ \, )$ be a *d*-topological space and $A \subseteq X$ is *d*-*open* then $A^{c}$ is *d*-*closed*.

### **Definition 3.4**

Let $( X,\mathcal {U},<\!\!\!\!\!\circ \, )$ be a *d*-topological space and $A\subseteq X.$ A point *x* in *A* is called an *interior point* of *A* if $x<\!\!\!\!\!\circ \, A.$

### *Remark*

Interior point of *A* is an *open set*.

### **Definition 3.5**

Let $( X,\mathcal {U},<\!\!\!\!\!\circ \, )$ be a *d*-topological space and $A\subseteq X.$ A point *x* in *X* is said to be *limit point* of *A* if for every $U\in \mathcal {U}_{x}$ there exist $y\ne x$ in *A* such that $y<\!\!\!\!\!\circ \, U.$

### **Definition 3.6**

Let (*X*, *d*) be a *d*-metric space and $f : X \rightarrow X$. If there is a number $0 < \alpha < 1$ such that $d(f(x), f(y)) \le \alpha d(x, y) \forall x, y \in X$ then *f* is called a *contraction*.

### **Definition 3.7**

( Sarma and Kumari 2012) Let (*X*, *d*) be a *d*-metric space and $f:X\rightarrow X$ be a mapping. Write $V(x)=d(x,f(x))$ and $Z(f)=\{x/V(x)=0\}$. We call points of *Z*(*f*) as *coincidence point* of *f*. Clearly every point of *Z*(*f*) is a fixed point of *f* but the converse is not necessarily true.

### **Theorem 3.8**

*A subset*$F\subseteq X$*is said to bed*-*closediff a net*$(x_{\gamma })$*inFd*-*convergestoxthen*$x\in F.$

### *Proof*

Suppose $F\subseteq X$ is *d*-closed.

Let $(x_{\gamma })$ be a net in *F* such that lim $d(x_{\gamma },x)=0.$

We shall prove that $x\in F.$

Let us suppose $x\notin F$ which implies that $x\in X-F,$ which is open.

Thus there exists $A\in \mathcal {U}_{x}$ such that $A\subset X-F.$

As $A\in \mathcal {U}_{x}$ there exists $\ \epsilon >0$ such that $\mathcal {B}_{\epsilon }(x)\subset A$.

Since lim $d(x_{\gamma },x)=0$ there exists $\gamma _{0}$ such that $d(x_{\gamma },x)< \epsilon$ for $\gamma \ge \gamma _{0}$.

Hence $$ x_{\gamma }\in \mathcal {B}_{\epsilon }(x)\subset A\subset X-F.$$ A contradiction.

It follows that $x\in F.$

Conversely, assume that if a net $(x_{\gamma })$ in *Fd*-*converges* to *x* then $x\in F.$

We shall prove that $F\subseteq X$ is *d*-closed.

$${\rm i.e} \ X-F$$ is *d*-open. 

For this we have to prove that for every $x\in X-F$ there exists $A\in \mathcal {U}_{x}$ such that 

$$A\subseteq X-F.$$

Suppose for some $x\in X-F$ there exists $A\in \mathcal {U}_{x}$ such that $A\nsubseteq X-F.$

Let $$ x_{A}\in A-(X-F).$$

As $\mathcal {U}_{x}$ is a direct set under set inclusion $A\le B \ if \ B\subseteq A.$

Thus $\{x_{A}/A\in \mathcal {U}_{x}\}$ is a net.

Let $$\epsilon >0, \ A_{0}=\mathcal {B}_{\epsilon }(x)\in \mathcal {U}_{x}$$.

If $$A\ge A_{0} \ ,A\subseteq A_{0}, $$

Thus $x_{A}\in A_{0}$ implies that $d(x_{A},x)<\epsilon$.

It follows that lim$d(x_{A},x)=0$.

Which implies that $x\in F.$ A Contradiction.

So for all $x\in X-F$ there exists $A\in \mathcal {U}_{x}$ such that $A\subseteq X-F.$

Which completes the proof. $\square$

### *Remark*

For each $\delta >0,\mathcal {B}_{\delta }(x)$ is a *d*-neighborhood of *x*.

### **Theorem 3.9**

*Let*$( X,\mathcal {U},<\!\!\!\!\!\circ \, )$*be ad-topological space and let*$\mathcal {U}_{x}$*be the collection of all subsetsUofXsuch that*$x<\!\!\!\!\!\circ \, U.$*Then*$\mathcal {U}_{x}$*is said to be a basis for a topology onXif*

(i) *For each*$x\in X,$*there exists*$U\in \mathcal {U}_{x}$*such that*$x<\!\!\!\!\!\circ \, U.$
(ii) *If*$x<\!\!\!\!\!\circ \, U_{1}\cap U_{2}$*there exists*$U_{3}\in \mathcal {U}_{x} \,{{such \, that}}\, x<\!\!\!\!\!\circ \, U_{3}$*and*$U_{3}\subseteq U_{1}\cap U_{2}.$

### *Proof*

(*i*) is clear.

Since $$x<\!\!\!\!\!\circ \, U_{1}\cap U_{2}$$ implies $$U_{1}\cap U_{2}\in \mathcal {U}_{x}.$$

So there exists $\epsilon >0$ such that $\mathcal {B}_{\epsilon }(x)\subset U_{1}\cap U_{2}.$

Since balls are *d*-neighborhood, choose $U_{3}=\mathcal {B}_{\frac{\epsilon }{2}}(x)\in \mathcal {U}_{x}.$

Then $$x<\!\!\!\!\!\circ \, U_{3}$$ and $$\mathcal {B}_{\frac{\epsilon }{2}}(x)\subset U_{1}\cap U_{2}.$$$$\square $$

### **Lemma 3.10**

*LetXbe any set and*$\mathcal {B},\mathcal {B}^{\prime }$*be basis for the topologies*$\mathfrak {J}$*and*$\mathfrak {J}^{\prime }$*respectively. Then the following are equivalent.*

(i) $$\mathfrak {J}^{\prime }$$*finer than*$$\mathfrak {J}$$$$(\mathfrak {J}\subseteq \mathfrak {J}^{\prime })$$
(ii) ${For \, each}\,  x\in X$*and each basis element*$B\in \mathcal {B}$*with*$x\in B$*there exists a basis element*$B^{\prime }\in \mathcal {B}^{\prime }$*such that*$x\in B^{\prime }$*and*$B^{\prime }\subseteq B.$

### **Theorem 3.11**

*Let*$(X,d,\mathfrak {J})$*be the topology induced from thed-topological space*$( X,\mathcal {U},<\!\!\!\!\!\circ \, )$*obtained fromd-metric as in Proposition 2.4,*$\mathfrak {J}_{d}$*be the topology induced by thed-metric then*$\mathfrak {J}=\mathfrak {J}_{d}.$

### *Proof*

Let $\mathcal {V}_{\epsilon }(x)=\mathcal {B}_{\epsilon }(x)\cup \{x\}.$ Then the collection $\mathfrak {B}=\{\mathcal {V}_{\epsilon }(x)/x\in X\}$ is a basis for $\mathfrak {J}_{d},$ and $\mathcal {U}_{x}=\{U\subset X /x <\!\!\!\!\!\circ \, U\}$ is a basis for $\mathfrak {J}.$ Clearly $\mathfrak {J}_{d}\subset \mathfrak {J},$ since $\mathcal {V}_{\epsilon }(x)$ is a *d*-neighborhood.

Let $x\in X$ and $U\in \mathcal {U}_{x}$ such that $x\in U.$

Since $x<\!\!\!\!\!\circ \, U$ there exists $\epsilon >0$ such that $\mathcal {B}_{\epsilon }(x)\subseteq U.$

Which implies $$\{x\}\cup \mathcal {B}_{\epsilon }(x)\subseteq U$$.

So $$\mathcal {V}_{\epsilon }(x)\subseteq U.$$ Hence $$\mathfrak {J}\subset \mathfrak {J}_{d}.$$$$\square $$

### **Theorem 3.12**

*Let*$( X,\mathcal {U},<\!\!\!\!\!\circ \, )$*be ad-topological space and*$A\subseteq X$*and*$x\in X$*the following are equivalent, assume*$\mathcal {B}_{\epsilon }(x)\ne \phi$*for every*$\epsilon >0.$

1. *There exists*$(x_{n})\in A$*such that lim*$d(x_{n},x)=0$
2. *For every*$U\in \mathcal {U}_{x}$*there exists*$\ y\ne x$*inAsuch that*$y<\!\!\!\!\!\circ \, U.$

### *Proof*

Let $U\in \mathcal {U}_{x}$ there exists $\epsilon >0$ such that $\mathcal {B}_{\epsilon }(x)\subseteq U.$

Since (1) holds, lim$d(x_{n},x)=0$.

Which implies that, there exists *N* such that $$d(x_{n},x)< \epsilon $$$$\forall n\ge N.$$

Let $y=x_{N}$ and $r=\epsilon -d(x_{N},x)$ then $\mathcal {B}_{r}(y)\subset \mathcal {B}_{\epsilon }(x)\subset U$.

It follows that $\mathcal {B}_{r}(y)\subset U.$

So $y<\!\!\!\!\!\circ \, U.$ Hence (2) holds.

Assume that (2) holds. Let $U=\mathcal {B}_{\frac{1}{n}}(x),$ there exists $x_{n}\ne x$ in *A* such that $x_{n}<\!\!\!\!\!\circ \, U=\mathcal {B}_{\frac{1}{n}}(x).$

*i*.*e* there exists $\epsilon _{n}<\frac{1}{n}$ such that $\mathcal {B}_{\epsilon _{n}}(x_{n})\subset \mathcal {B}_{\frac{1}{n}}(x).$

Let $$y_{n}\in \mathcal {B}_{\epsilon _{n}}(x_{n})$$.

Which implies that $d(x_{n},y_{n})<\frac{1}{n}$ and $d(x,y_{n})<\frac{1}{n}$.

Hence $$d(x_{n},x)<d(x_{n},y_{n})+d(y_{n},x)<\frac{2}{n}$$.

Which yields lim $d(x_{n},x)=0.$ Hence (1) holds. $\square$

### **Theorem 3.13**

*Let*$( X,\mathcal {U},<\!\!\!\!\!\circ \, )$*be thed-topological space obtained fromd-metric*$(X,\varrho )$*as in Proposition 2.4 .Then balls ared-open.*

### *Proof*

Let $\mathcal {B}_{\epsilon }(x)$ be a ball with center at *x* and radius $\epsilon$.

It sufficies to prove that $\mathcal {B}_{\epsilon }(x)$ is *d*-open.

*i*.*e* we shall prove for every $y \in \mathcal {B}_{\epsilon }(x)$ there exists $U\in \mathcal {U}_{y}$ such that $U\subset \mathcal {B}_{\epsilon }(x).$

Since $$y\in \mathcal {B}_{\epsilon }(x)$$ implies $$ d(x,y)<\epsilon $$.

Choose $$\delta =\epsilon -d(x,y)$$.

As $\mathcal {B}_{\delta }(y)$ is a *d*-neighborhood, now let $U=\mathcal {B}_{\delta }(y)$.

So it is sufficient to prove that $U\subset \mathcal {B}_{\epsilon }(x)$.

Let $$z\in U$$.

This implies that $d(y,z)<\delta <\epsilon -d(x,y)$.

Then $$ d(x,z)<\epsilon $$.

It follows that $z\in \mathcal {B}_{\epsilon }(x)$.

Hence $$U\subset \mathcal {B}_{\epsilon }(x).$$$$\square $$

### **Theorem 3.14**

*Let*$( X,\mathcal {U},<\!\!\!\!\!\circ \, )$*be ad-topological space obtained fromd-metric*$(X,\varrho )$*as in Proposition 2.4. Then*$(X,d,\mathfrak {J})$*is a Haussdorff space.*

### *Proof*

Suppose $$x\ne y\Rightarrow d(x,y)>0$$.

Let us choose $\delta =d(x,y).$

Let $\mathcal {B}_{\frac{\delta }{2}}(x),\mathcal {B}_{\frac{\delta }{2}}(y)$ be the *d*-neighborhoods of *x* and *y* respectively.

It sufficies to prove $\mathcal {B}_{\frac{\delta }{2}}(x)\cap \mathcal {B}_{\frac{\delta }{2}}(y)=\phi$.

Let $$z\in \mathcal {B}_{\frac{\delta }{2}}(x)\cap \mathcal {B}_{\frac{\delta }{2}}(y)$$.

Which implies that $z\in \mathcal {B}_{\frac{\delta }{2}}(x)$ and $z\in \mathcal {B}_{\frac{\delta }{2}}(y)$.

So $$ d(x,z)< \frac{\delta }{2}$$ and $$d(y,z)< \frac{\delta }{2}$$.

It follows $$ d(x,y)< \delta =d(x,y)$$.

Which is a contradiction. $\square$

### **Theorem 3.15**

*Let*$( X,\mathcal {U},<\!\!\!\!\!\circ \, )$*be ad-topological space obtained formd-metric*$(X,\varrho )$*as in Proposition 2.4. Then singleton sets ared-closed in*$(X,d,\mathfrak {J}).$

### *Proof*

Let $x\in X$, we have to prove that $\{x\}$ is *d*-closed or it is sufficies to prove $X-\{x\}$ is *d*-open.

*i*.*e* for each $y\in X-\{x\}$ there exists $U\in \mathcal {U}_{y}$ such that $U\subseteq X-\{x\}.$

Since $$y\ne x,$$ implies $$ d(x,y)>0$$.

Which yields $$x\notin \mathcal {B}_{\epsilon }(y)$$.

Thus, there is a *d*-neighborhood,$\mathcal {B}_{\epsilon }(y)\in \mathcal {U}_{y}$ such that $\mathcal {B}_{\epsilon }(y)\subseteq X-\{x\}$.

Hence $\{x\}$ is *d*-closed. $\square$

### **Corollary 3.16**

*Let*$( X,\mathcal {U},<\!\!\!\!\!\circ \, )$*be ad-topological space obtained formd-metric*$(X,\varrho )$*. Then*$(X,d,\mathfrak {J})$*is a*$T_{1}$*-space*.

### **Corollary 3.17**

*Let*$(X,d,\mathfrak {J})$*be ad-topological space. Then the collection*$\{\mathcal {B}_{\epsilon }(x)/x\in X\}$*is an open base atxforX*.

## Main theorems

Wardowski (2012) introduced a new type of contraction called F-contraction and proved a new fixed point theorem concerning F-contraction and supported by computational data illustrate the nature of F-contractions. In this section, we present a theorem which generalizes the Wardowski’s theorem.

### **Definition 4.1**

(Wardowski 2012) Let $F:R^{+}\rightarrow R$ be a mapping satisfying,

(i) *F* is strictly increasing, i.e for all $\alpha ,\beta \in R^{+}$ such that $\alpha <\beta ,F(\alpha )<F(\beta )$
(ii) For each sequence $\{\alpha _{n}\}_{n\in \mathbb {N}}$ of positive numbers $lim_{n\rightarrow \infty }\alpha _{n}=0$ iff $lim_{n\rightarrow \infty }F(\alpha _{n})= -\infty$
(iii) There exists $k\in (0,1)$ such that $lim_{\alpha \rightarrow 0^{+}}\alpha ^{k}F(\alpha )=0$

A mapping $$T:X\rightarrow X$$ is said to be an F-contraction if there exists $$\tau >0$$ such that for all $$x,y\in X, d(Tx,Ty)>0 \Rightarrow $$$$\tau +F(d(Tx,Ty))\le F(d(x,y)).$$

### **Theorem 4.2**

(Sgroi and Vetro 2013) *Let* (*X*, *d*) *be a complete metric space and let*$T:X\rightarrow X$*be an F-contraction thenThas a unique fixed point*$x^{*}\in X$*and for every*$x_{0}\in X$*a sequence*$\{T^{n}x_{0}\}_{n\in \mathbb {N}}$*is convergent to*$x^{*}.$

In the literature one can find some interesting papers concerning F-contractions; (see for example Cosentino and Vetro 2014; Sgroi and Vetro 2013; Secelean 2013; Paesano and Vetro 2014; Hussain and Salimi 2014).

### **Definition 4.3**

By $\mathcal {G}$ we denote the set of all monotone decreasing real functions $g:[0,\infty )\rightarrow [0,\infty ),$ such that $g(x)=0$ iff $x=0$ and $lim_{t\rightarrow 0^{+}}\ g(t)=0.$

### **Lemma 4.4**

*Let*$g\in \mathcal {G}$*and*$\{\epsilon _{n}\}\subseteq [0,\infty )$*, then from*$g(\epsilon _{n})\rightarrow 0$*it follows that*$\epsilon _{n}\rightarrow 0.$

### *Proof*

Routine. $$\square $$

### **Theorem 4.5**

*Let* (*X*, *d*) *be ad-metric space,*$x\in X,\{x_{n}\} \subseteq X$*and*$g\in \mathcal {G}$*satisfying subadditive property. Define*$d^{*}:X^{2}\rightarrow [0,\infty )$*by*$d^{*}(x,y)=g(d(x,y))$*for any*$x,y\in X.$*Then*

1. $(X,d^{*})$*is ad-metric space*.
2. lim$d(x_{n},x)=0$*iff* lim$d^{*}(x_{n},x)=0.$
3. (*X*, *d*) *is complete iff*$(X,d^{*})$*is complete*.

### *Proof*

Let $$d^{*}(x,y)=0$$.

Which yields $$ g(d(x,y))=0$$ implies $$ d(x,y)=0.$$

So $$ x=y.$$$$d^{*}(x,y)=d^{*}(y,x)$$ follows from $$g(d(x,y))=g(d(y,x)).$$

Now consider $$d^{*}(x,z)=g(d(x,z))$$

$$\le g(d(x,y)+d(y,z))$$

$\le g(d(x,y)+g(d(y,z))$ since *g* is subadditive.

$$=d^{*}(x,y)+d^{*}(y,z)$$.

It follows that $d^{*}(x,z)\le d^{*}(x,y)+d^{*}(y,z)$.

Hence $d^{*}$ is a *d*-metric. This completes the proof of (1).

Let lim$d(x_{n},x)=0$. It follows that lim$gd(x_{n},x)=g(0)=0.$

Which implies lim$d^{*}(x_{n},x)=0.$

Suppose lim$d^{*}(x_{n},x)=0.$ By above lemma, it follows that lim$d(x_{n},x)=0.$ Which completes the proof of (2).

Let us suppose that (*X*, *d*) is complete. Thus for every $\epsilon >0$ there exist $n_{1}\in N$ such that $d(x_{n},x_{m})<\epsilon$ for all $m,n\ge n_{1}$.

Which yields lim$d(x_{n},x_{m})=0$.

Which implies lim$d^{*}(x_{n},x_{m})={\text{lim}}gd(x_{n},x_{m})=g(0)=0$; because *g* is continuous at 0. So $\{x_{n}\}$ is a Cauchy sequence in $(X,d^{*}).$ By using (2), we get $(X,d^{*})$ is complete.

Conversely suppose that $(X,d^{*})$ is complete.

Let $\{x_{n}\}$ is a Cauchy sequence in $(X,d^{*}).$

Then for every $\epsilon >0$ there exist $n_{1}\in N$ such that $d^{*}(x_{n},x_{m})<\epsilon$ for all $m,n\ge n_{1}.$

Thus lim$$d^{*}(x_{n},x_{m})=0$$.

It follows that lim$gd(x_{n},x_{m})=0$.

By above Lemma, lim$d(x_{n},x_{m})=0$.

Which implies that $\{x_{n}\}$ is a Cauchy sequence in (*X*, *d*). By using (2) we conclude that (*X*, *d*) is complete. $\square$

### **Definition 4.6**

Let $F:R^{+}\rightarrow R$ be a mapping satisfying,

(i) *F* is strictly increasing, i.e for all $\alpha ,\beta \in R^{+}$ such that $\alpha <\beta ,\ F(\alpha )<F(\beta ).$
(ii) For each sequence $\{\alpha _{n}\}_{n\in \mathbb {N}}$ of positive numbers $lim_{n\rightarrow \infty }\alpha _{n}=0$ iff $lim_{n\rightarrow \infty }F(\alpha _{n})=-\infty$
(iii) There exists $$k\in (0,1)$$ such that $$lim_{\alpha \rightarrow 0^{+}}\alpha ^{k}F(\alpha )=0$$. A mapping $$T:X\rightarrow X$$ is said to be an $$\mathcal {G}F$$-contraction if there exists $$\tau >0$$ such that for all $$x,y\in X$$, $$g(d(Tx,Ty))>0 \Rightarrow $$$$\tau +F(g(d(Tx,Ty)))\le F(g(d(x,y))).$$

### **Theorem 4.7**

*Let* (*X*, *d*) *be a completed-metric spaceand let*$T:X\rightarrow X$*be an*$\mathcal {G}F$*-contraction. ThenThas a unique fixed point*.

### *Proof*

Define $d^{*}:X^{2}\rightarrow [0,\infty ]$ by $d^{*}(x,y)=g(d(x,y))$ for any $x,y\in X$ and $g\in \mathcal {G}.$ By lemma 4.4 and theorem 4.5 it follows that $(X,d^{*})$ is a *d*-metric space.

We have, when $d^{*}(Tx,Ty))>0$ implies $\tau +F(d^{*}(Tx,Ty))\le F(d^{*}(x,y)).$

Then by using same proof as in Theorem 4.2, we can conclude that *T* has a unique fixed point. $\square$

### **Theorem 4.8**

*Let*$(X,\rho )$*be a completed-metric space and let*$f:X\rightarrow X$*be a contraction and*$( X,\mathcal {U},<\!\!\!\!\!\circ \, )$*be thed-topological space obtained from*$(X,\varrho )$*. Thenfhas a unique coincidence point forf*.

### *Proof*

Let $$x_{0}\in X$$. Choose $$x_{n+1}=f(x_{n})=f^{n}(x_{0})$$.

Then $f^{n}(x_{0})$ is a cauchy sequence and converges in $(X,\varrho )$ to some point *u*.

*i*.*eu* = lim $f^{n}(x_{0}).$ Since *f* is a contraction it is also *d*-continuous, by Proposition 2.10, $f(u)=lim f^{n+1}(x_{0}).$

Hence $$ d(u,f(u))=lim_{n\rightarrow \infty }d(f^{n}(x_{0}),f^{n+1}(x_{0}))<lim_{n\rightarrow \infty }\alpha ^{n}d(x_{0},f(x_{0}))$$ = 0. Since $$0<\alpha <1$$, $$ d(u,f(u))=0$$.

Thus *u* is a coincidence point of *f*. $\square$

### Uniqueness

Let us suppose that *v* be the another coincidence point such that $d(v ,f(v ))=0$.

Thus $f(v )=v$ and $f(u )=u$. By using triangle inequality, $d(v ,u )\leq d(v ,f(v ))+d(f(v ),f(u ))+d(f(u ),u ) \leq \alpha d(v ,u )$ which implies $d(v ,u )=0$.

Hence $$u=v $$.
